# Modeling Hepatotropic Viral Infections: Cells vs. Animals

**DOI:** 10.3390/cells10071726

**Published:** 2021-07-08

**Authors:** Niloofar Khoshdel-Rad, Ensieh Zahmatkesh, Polina Bikmulina, Maria Peshkova, Nastasia Kosheleva, Evgeny A. Bezrukov, Roman B. Sukhanov, Anna Solovieva, Anastasia Shpichka, Peter Timashev, Massoud Vosough

**Affiliations:** 1 Department of Stem Cells and Developmental Biology, Cell Science Research Center, Royan Institute for Stem Cell Biology and Technology, ACECR, Tehran 1665659911, Iran; niloofarkhoshdelrad@yahoo.com (N.K.-R.); ensieh_zahmatkesh@yahoo.com (E.Z.); 2 Department of Regenerative Medicine, Cell Science Research Center, Royan Institute for Stem Cell Biology and Technology, ACECR, Tehran 1665659911, Iran; 3 Institute for Regenerative Medicine, Sechenov First Moscow State Medical University, 119991 Moscow, Russia; polina_bikmulina@mail.ru (P.B.); maria_peshkova@rambler.ru (M.P.); ana-shpichka@yandex.ru (A.S.); 4 World-Class Research Center “Digital biodesign and personalized healthcare”, Sechenov First Moscow State Medical University, 119991 Moscow, Russia; n_kosheleva@mail.ru; 5 FSBSI ‘Institute of General Pathology and Pathophysiology, 125315 Moscow, Russia; 6 Department of Urology, Sechenov First Moscow State Medical University, 119991 Moscow, Russia; eabezrukov@rambler.ru (E.A.B.); rb_suhanov@mail.ru (R.B.S.); 7 Department of Polymers and Composites, N.N. Semenov Federal Research Center for Chemical Physics, Russian Academy of Sciences, 119991 Moscow, Russia; ann.solovieva@gmail.com; 8 Chemistry Department, Lomonosov Moscow State University, 119991 Moscow, Russia

**Keywords:** animal models, hepatitis, hepatotropic virus, cell culture

## Abstract

The lack of an appropriate platform for a better understanding of the molecular basis of hepatitis viruses and the absence of reliable models to identify novel therapeutic agents for a targeted treatment are the two major obstacles for launching efficient clinical protocols in different types of viral hepatitis. Viruses are obligate intracellular parasites, and the development of model systems for efficient viral replication is necessary for basic and applied studies. Viral hepatitis is a major health issue and a leading cause of morbidity and mortality. Despite the extensive efforts that have been made on fundamental and translational research, traditional models are not effective in representing this viral infection in a laboratory. In this review, we discuss in vitro cell-based models and in vivo animal models, with their strengths and weaknesses. In addition, the most important findings that have been retrieved from each model are described.

## 1. Introduction

The lack of an appropriate platform for a better understanding of the molecular basis and the absence of reliable models to identify novel therapeutic agents for a targeted treatment are the two major obstacles for launching efficient clinical protocols in different types of viral hepatitis [1,2]. The hepatotropic viruses A, B, C, D, and E, are the most common causes of viral infections that can lead to liver failure. Hepatitis B virus (HBV), hepatitis C virus (HCV), and hepatitis D virus (HDV) are normally transmitted through organ transplants, transfusions, sex, and injections [3]. The HBV genome is a double-strand DNA, and this virus is classified into the genus of *Orthohepadnavirus* and the family of *Hepadnaviridae*. HCV is a small virus (55–65 nm in size) with an RNA genome and is categorized into the genus of *Hepacivirus* and the family of *Flaviviridae* [4]. HDV is a small and uncommon human pathogen containing a single-stranded circular RNA genome. This pathogen is classified into the genus of *Deltaviridae*. HDV has hepatitis B surface antigen (HBsAg), which surrounds the genomic RNA-nucleoprotein complex, thereafter, requires HBV to complete its life cycle and replicate [5,6] HBV, HCV, and HDV induce chronic liver inflammation that can finally result in cirrhosis and hepatocellular carcinoma [5,7]. HAV (Hepatitis A virus) and HEV (Hepatitis E virus) are generally transmitted through water or food [8]. The studies conducted in many countries have demonstrated that HAV is the most common type causing acute viral hepatitis. HAV is a single strand of plus sense RNA and belongs to the genus of *Hepatovirus* in the *Picornaviridae* family, and it is transmitted through the fecal-oral route [9]. HEV is classified into the genus of *Orthohepenvirus A* and the family of *Hepeviridae.* HEV is a nonenveloped positive-strand RNA virus that generally causes acute infections. Clinical observations indicate that HEV can lead to chronic infections in immunocompromised patients, including transplant recipients [8,10]. On the other hand, it is difficult to develop therapeutics for these viral hepatites, due to the lack of a reliable model. Therefore, establishing a defined and suitable model as a platform for studying hepatotropic viruses is necessary [11]. Studying hepatotropic viruses in vivo is limited because most of them are species-specific. For instance, HBV and HCV infect only humans, tree shrews, and some nonhuman primates [2,12]. Thus, the most promising approach to study human hepatotropic viruses is using genetically modified animals. In parallel, cell-based systems have received remarkable attention in hepatotropic viral infection modeling. Monolayer and three dimensional (3D) culture systems have been used for understanding the molecular basis of hepatotropic viruses and evaluating novel antiviral agents; however, they did not help the scientific community in understanding some aspects of viral pathogenesis, including the differences between the genotypes of hepatotropic viruses. In this review, we discuss different in vitro and in vivo model systems for the study of hepatotropic viruses, and their advantages and disadvantages.

## 2. In Vitro Models

### 2.1. A monolayer Culture (Primary Cells, Cell Lines, and Coculture System)

Primary human hepatocytes (PHH), as the most authentic cell culture model for hepatotropic viruses, were used in many studies [13,14,15]. For in vitro HBV studies, PHH have been used as a gold standard platform in cell culture for many years, and many studies used PHH as a model for the HBV and HCV infection [16,17]. A later study indicated that PHH also supported an HEV infection with a Kernow-C1/p6 strain [18]. Other studies demonstrated that PHH were susceptible to HDV [19,20,21]. Taylor et al. provided the first evidence that purinergic receptor functionality was essential for the process of PHH infection by HDV and HBV [22]. Since the source of chronic HEV infection is unknown, some studies explored extrahepatic sources for HEV replication. In 2019, a study [23] introduced human polarized enterocytes (primary intestinal cells) as a model for HEV replication. Moreover, El-Mokhtar and his colleagues demonstrated that primary human endometrial stromal cells were susceptible and permissive to HEV infection, and this type of primary cells could be an endogenous source of HEV infection during pregnancy and mediate the HEV vertical transmission [24]. However, their utility is hampered by some shortcomings, including the viability of donated cells, availability, and limitation for a long term ex vivo culture [25]. These challenges have led to establishing various culture models [13].

To preserve the liver-specific function and extend the lifetime of PHH, some studies employed a coculture strategy that consisted of PHH and supportive stromal cells (murine fibroblast cells) with the ratio of PHH and supportive cells estimated as 1:4. In this micropatterned coculture system, the phenotype and functionality of PHH were maintained for more than several weeks, and the results showed a robust infection of PHH with HCV [26,27]. In consistency with the previous studies, March and collaborators established a micropatterned coculture system in which islands of PHH in a 2D culture were surrounded by fibroblast cells. The results demonstrated that this culture system was suitable for the study of HBV and HCV colonization and replication [28]. Furthermore, Zhou and colleagues applied a coculture system that consisted of fetal PHH and liver nonparenchymal cells for prolonged susceptibility to the HBV infection [29]. Winer and collaborators, by self-assembling of PHH and mouse stromal cells in a coculture system, provided a scalable platform for long-term colonization and replication of HBV [30]. Later, this group demonstrated that self-assembly of PHH with nonparenchymal mouse embryonic fibroblast 3T3-J2 cells in a coculture system is a versatile platform for studying HBV/ HDV coinfections and holds great promise for performing chemical library screens and improving our understanding of the host response to such infections [31]. Furthermore, in 2020, a study [32] reported that peripheral blood mononuclear cells and bone marrow-derived macrophages from healthy donors were susceptible to HEV in vitro. Since renal diseases are associated with the HEV infection, in 2020, a study [33] reported a possible mechanism for HEV-mediated renal disease. The authors isolated CD10+/CD13+ primary proximal tubular epithelial cells, infected them in vitro with HEV inoculum, and then the expression of inflammatory and kidney injury markers was assessed in cocultivation with/without immune cells isolated from the same donors. The results demonstrated that coculture of immune cells with HEV-infected epithelial cells exacerbated the inflammatory response and induced kidney injury [33]. However, applying the coculture system for PHH could not overcome the drawbacks of the broad usage of PHH.

Some studies reported establishing two hepatoma cell lines named HepAD38 and HepDE19, which expressed HBV pgRNA under the control of the tetracycline-repressible promoter instead of the native viral core promoter [34,35]. A Chinese group introduced a new hepatoma HLCZ01 cell line which supported the whole life cycle of both HBV and HCV. The results indicated that this cell line provided a powerful tool for supporting the accurate life cycle of the virus with a normal genetic background [36]. Since the HBV infection also occurs at extrahepatic sites, the identification of the relevant host factor in nonhepatic cells is essential. Yang and collaborators reconstituted the HBV infection in the human embryonic kidney (HEK) 293T cells by exogenous expression of the nuclear hormone receptors HNF4α, RXRα, and PPARα, and the HBV receptor, sodium taurocholate cotransporting polypeptide (NTCP). Their results suggested that these factors could play a pivotal role in the HBV infection of nonhepatic cells [37]. A German group established a stable new cell line named HepNB2.7, which was susceptible to HDV and supported the full viral life cycle [38].

Several polarized human liver cell lines were produced, such as HepaRG and HepG2 [39]. The hepatoma HepaRG cell line, a bipotent liver progenitor cell line, upon induction by dimethyl sulfoxide (DMSO), was permissive to HBV [40]. To optimize the in vitro differentiation of HepaRG, Yuan and his colleagues used four small molecules (FPH1, FPH2, FH1, and XMU-MP-1) and increased both the hepatic differentiation and proliferation capacity of HepaRG cells in vitro and in vivo, which is essential for the HBV infection [41]. However, using this cell type has some drawbacks, including a low viral yield and replication, a lack of covalently closed circular DNA (cccDNA) amplification, and difficulty understanding the HBV life cycle [42]. Sophie Roge’e and her colleagues introduced the HepaRG cell line and PICM19, derived from the primary culture of pig embryonic stem cells as in vitro models for HEV replication. They reported that these in vitro culture systems support HEV replication and release of encapsulated RNA [43]. In accordance with this study, Pellerin and her colleagues showed that HepaRG is a relevant and efficient in vitro model of HEV replication that could be used to study HEV and identify effective antiviral drugs against chronic HEV infection [44]. Besides, another study introduced a new procedure using a cocktail of 5 chemicals (Forskolin, SB431542, IWP2, DAPT, and LDN193189), allowing fast differentiation and efficient HDV-infection of HepaRG cells [45]. Many studies applied the HepaRG cell line as a unique model to study the interplay between HBV/HDV and hepatocyte-specific innate immunity, as well as to explore new therapeutic developments [46,47].

HepG2 [(Hepatoma G2); derived from a hepatoblastoma] and Huh7 [(human hepatocellular carcinoma cell line 7); derived from a hepatocellular carcinoma] are two human hepatoma cell lines which are widely used in antiviral studies, especially those regarding HBV. These cell lines support the virus replication when transfected with HBV [48,49]. Some studies indicated that HepG2 and Huh-7 cell lines as NTCP-expressing lines could be efficiently infected with HBV and HDV [47,50]. Another study showed altered gene expression in HepG2 cells induced by HBV and HCV, which provides new insight into the mechanism of HBV and HCV infection and improves the understanding of the differences in the molecular pathogenesis of HBV and HCV [51]. Not long ago, it has been found that HepG2-NTCP cells are hardly infected with HBV-positive sera and that a clonal section is needed to recognize clones producing high titers of infectious progeny [52]. Recently, Kempp et al. established a stable cell line (HepNB2.7) by transducing HepG2 cells with genes encoding the NTCP-receptor and the HBV envelope proteins that support the full viral life cycle of HDV and HBV [38]. Furthermore, to support HEV replication, in a study [53] HepG2 and Huh-7 lines were used. A study [54] reported a simple yet robust cell culture HEV infection method. The model was based on the HEV genotype 3 Kernow-C1 p6 strain and the two human hepatoma cell lines (HepG2 and HepG2/C3A) combined with various media conditions.

To investigate various viruses’ interactions, Jian and collaborators developed a scalable and visualizable HAV/HCV coinfection model in Huh-7 cells. Their finding revealed that the simultaneous presence of HAV-HCV did not affect the viral RNA synthesis of both viruses. They suggested that indirect interactions may lead to the suppression and clearance of HCV in HAV/HCV coinfected patients [55]. Sun and colleagues reported the creation of stable TetOFF hepatoma cell lines (HepG2 and Huh7) to control HBV production. Their approach presented some advantages, including applying both hepatoma cell lines and using a two-step procedure rather than cotransfection [56]. König and collaborators tried to develop a perfect cell culture platform for HBV amplification from clinical specimens. To achieve this, they applied slow proliferating HepG2- NTCP for the HBV infection. The obtained results demonstrated that this cell line successfully supported the whole HBV life cycle, as well as long-term amplification of HBV [52]. However, these cell lines could do mediate the early-stage virus infection, such as the entry, uncoating, and the formation of cccDNA [25]. Besides, the causes of limited HCV permissiveness in cell lines are not completely understood, but the most important aspects have been identified. Restricted expression of cell surface receptors, such as CD81 and scavenger receptor class B type I (SRBI), is recognized to be associated with the restriction of the HCV entrance [25,57,58].

Therefore, to better mimic the viral life cycle and host-virus interactions, more representative and functional cell types are urgently needed. Pluripotent stem cells (PSCs) are a renewable source of cells and can be obtained via different protocols [59]. Since the cells derived from PSCs would be functional and similar to primary cells, they may be suggested as an ideal replacement for currently used cell-based models [60]. In 2012 and 2015, 2D cultures of HLCs derived from human PSCs were shown to support the entry and replication of HCVcc [61,62]. In 2017, these findings were corroborated by Yan et al. [63]. Various host factors are critical to the HBV infection. Hepatic-like cells (HLCs) mostly resemble PHH, due to the high expression of crucial factors for the HBV infection and replication. As a first confirmation, Shlomai and colleagues indicated that PSCs-derived HLCs were permissive to the HBV infection [27]. Consistent with the previous study, two other studies demonstrated that stem cell-derived HLCs could completely support the HBV infection for about one month [25,63,64]. Moreover, several studies reported that PSCs-derived HLCs could successfully support various forms of the HCV infection [65,66]. Some studies have successfully demonstrated that the complete replication cycle of HEV is supported by iPSC-derived HLCs [67,68,69]. In cell-based models of liver disease, researchers understand the importance of hepatocyte polarity. Because these viruses enter hepatocytes through the basolateral membrane, the hepatocyte polarity is crucial for the productive entry of hepatitis viruses [70]. In one study [70], researchers differentiated hPSCs into columnar polarized HLCs using transwell filters. These HLCs secreted urea, albumin, and lipoproteins basolaterally, while bile acids were produced apically. The authors showed that polarized HLC supported HEV infection and replication, and mimicked fundamental steps of the natural infectious cycle in vivo.

Nevertheless, although great efforts are directed toward improving differentiation protocols to achieve better maturation, little progress has been achieved so far. Therefore, there is a need to provide a suitable niche that is more similar to the in vivo architecture, such as spheroids, organoids, bioprinted microtissues, and microfluidic chip devices [60].

### 2.2. Three Dimensional (3D) Culture Systems

Cell polarity, micro-patterned complexity, and cellular interactions are absent in 2D culture systems. Because of the drawbacks of 2D model systems, researchers are looking for alternative three-dimensional (3D) models. The created 3D models, including spheroids, organoids with multicellular structures, and their specific extra-cellular matrix (ECM), were shown to exhibit higher tissue-specific microenvironmental complexity, more mature cells, and better physiological functionality compared to their simple 2D counterparts [71]. Additionally, other cell culture models, including cells embedded in a specific scaffold and single-channel microfluidic devices, are promising platforms for in vitro models to study hepatotropic viruses [72].

#### 2.2.1. Spheroid Culture Models

3D liver spheroids are multicellular aggregates that exhibit complex cellular communications and polarity in vitro and have characteristics that resemble complex native tissues. These 3D models have been developed through different approaches, such as scaffold-free and scaffold-based 3D cultures, and may be created from primary hepatocytes, established cell lines, and hPSCs-derived cells [73].

Several studies have established liver spheroid models to study a hepatotropic virus’s life cycle in the liver tissue. Chong and colleagues generated primary human hepatocyte spheroids from uninfected liver resections. Spheroids were inoculated with an HCV-positive serum. The obtained data showed that spheroids had a differentiated phenotype and expressed putative HCV receptors. Furthermore, the HCV RNA was detected in the cells, as well as in the supernatant of the culture media [73]. Aly and colleagues established a 3D model of an immortalized primary human hepatocyte cell line (HuS-E/2 cells) in a thermoreversible polymer that had a higher susceptibility to the infection and replication of HCV, compared to 2D models. Unlike recombinant HCV models, which were not effective in developing anti-HCV drugs, the natural HCV models are relatively polymorphic and may be useful in new drug development projects in personalized medicine [74].

In a study by Sainz and colleagues, Huh7 cells were cultured in a 3D rotating wall vessel to generate multilayered 3D liver aggregates. These 3D structures expressed high levels of Phase I and Phase II related xenobiotic drug metabolism genes, as well as hepatocyte-specific transcripts compared to the Huh7 monolayer culture. The data showed increased expression and organization of different proteins composing cell adhesion and tight junction proteins, polarity-related proteins, and HCV receptors. Thus, these 3D Huh7 aggregates could be a model to study the HCV infection effects on the function of liver cells [75]. Murakami and colleagues showed that a dicistronic genome-length Con1 HCV RNA of genotype 1b supported the synthesis and secretion of infectious HCV particles in Huh-7 spheroids in a thermoreversible gelation polymer. The HCV particle size and morphology in these polarized liver spheroids resembled virus-like particles detected in the sera of patients with hepatitis C. This 3D culture system was used to study the viral morphogenesis and the biophysical properties of HCV particles in a natural host microenvironment, and it provided a valuable tool for the evaluation of anti-HCV drugs [76]. Molina-Jimenez and colleagues developed a Matrigel-embedded spheroid model of Huh-7 cells to study the HCV infection and virion formation. This 3D liver spheroid model had hepatocyte-like polarized features and generated a continuous network of functional proto-bile canaliculi structures. These Matrigel-embedded 3D spheroids were susceptible to the HCV infection and generated viral particles with specific infectivity [77]. The use of the Matrigel-embedding method for liver spheroid formation may lead to dysregulation of some signaling pathways and gene expression [78]. In another study, 3D spheroids of Huh 7.5 cells and primary human hepatocytes were created in a galactosylated cellulosic sponge. These spheroids had a uniform size and polarized structures, as well as higher expression and localization of all the HCV-specific entry proteins. This model improved the liver-specific functions and viral entry compared to conventional 2D cultures [79].

Fu and colleagues dedifferentiated human primary hepatocytes into expandable liver progenitor-like cells (HepLPCs) using certain chemical agents. These HepLPCs could be redifferentiated into functional hepatocytes. The obtained data showed that redifferentiation in the spheroid culture conditions resulted in upregulation of the viral entry receptor, NTCP, compared to 2D culture. These liver spheroids have the potential to support the HBV infection and secreted newly produced virions. Furthermore, the researchers have shown that a reverse transcriptase inhibitor, entecavir, and the NTCP substrate, tauroursodeoxycholic acid, block HBV binding in liver spheroids and act as a suitable platform for the study of host interactions with HBV and antiviral drugs [80].

The chronic HBV infection is associated with a high rate of hepatocellular carcinoma (*HCC*) development. Song and colleagues established HBV DNA-secreting HCC cell lines (AMC-H1 and AMC-H2) from infected patients. Then, they generated patient-derived 3D multicellular tumor spheroids consisting of HBV-infected HCC cells, fibroblasts, human umbilical vein endothelial cells (HUVECs), and human hepatic stellate cells to screen potentially effective treatment strategies for precision cancer medicine. The data demonstrated that these multicellular tumor spheroids exhibited a clear selective response to anticancer drugs as opposed to homogeneous HCC spheroids. The use of a coculture system in spheroid formation is one step forward to the native tissue environment [81].

Most of the liver spheroid models to study the hepatotropic viruses have relied on the HBV and HCV infection, and liver spheroids for the hepatotropic viruses A, D, and E are still under development. Hereafter, liver spheroids could be used for modeling individual aspects of these hepatotropic viruses.

#### 2.2.2. Organoid Systems

Liver organoids are multicellular structures that can mimic the complex microenvironment of the liver tissue during development and diseases. These 3D models can be created using different cells and methods and provide a valuable research model for studying the mechanisms of viral pathogenesis, personalized infection pattern, and precision medicine [82,83,84].

Nie and colleagues used a coculture system of hiPSC-derived endoderm, HUVECs, and mesenchymal stem cells in 3D microwells to assess the liver organoids’ potential for the HBV infection and virus-host interactions. The cells were self-organized and differentiated into functional liver organoids. Then, the organoids were infected with HBV. Compared to with human iPSC-derived 2D hepatic-like cells, the liver organoids exhibited a better functionality and a higher susceptibility to the HBV infection. Moreover, the organoids could sustain HBV propagation and produce an infectious virus for up to 20 days. The HBV infection decreased the expression of hepatic-specific genes and increased the concentration of early biomarkers for acute liver failure, alanine aminotransferase (ALT), and lactate dehydrogenase (LDH), in the supernatant of infected organoids. The *iPSCs*-derived *3D* liver organoids had the advantage of providing the HBV infection models for precision medicine [85]. Crignis and colleagues developed liver organoids from healthy donors and HBV-infected patients. HBV-infected liver organoids displayed an aberrant gene expression signature highly associated with the expression pattern in the HCC cohort. Liver organoids derived from healthy donors were infected with both synthetic recombinant viruses and with HBV-positive patient serum. In both groups, HBS Ag and HBV-specific core proteins, as well as HBV virions, were detected in the culture supernatant. The infection and replication of HBV were increased significantly in the liver organoids under the differentiating culture conditions. The ex vivo infected liver organoids had a high susceptibility to the infection and replication of natural HBV, which was blocked by a nucleotide reverse transcriptase inhibitor, Tenofovir. Thus, these organoids can be a reliable model for the HBV drug screening [86].

In another study, the researchers established a Huh-7.5 organoid system that exhibited a hepatocyte-like structure and expressed viral colonization molecules. They used a single-particle tracking method for studying the traffic processes of HCV in Huh-7.5 organoids. The data showed that HCV was localized in the basolateral membrane; then, its virions were accumulated at tight junctions of liver organoids via the actin-dependent mechanism. The EGF receptor (EGFR) is required for the particle internalization, but is not involved in guiding the colonization of HCV. This hepatoma organoid model has a higher susceptibility to HCV infection than that of the monolayer cultures [87]. In another study, this group showed that mCd302 and mCr1l proteins impaired the HCV entry kinetics in Huh-7.5 organoids disrupting the steps of the complex entry cascade. Thereafter, Cd302 and Cr1l restrict the HCV cross-species transmission to mice [88].

Kulsuptrakui and colleagues generated bi-potent stem cell organoids from healthy liver tissue. The cells were self-organized and differentiated into hepatocytes. Then, the human liver organoids were infected with HAV, and infection assays were performed. The obtained data indicated that pharmacological inhibition of the TRAMP-like complex using PAPD5 and PAPD7 small molecules reduced HAV RNA in human liver organoids. Thereafter, liver organoids provide a novel model for host-directed therapy of HAV infection [89].

The use of liver organoids to study the pathogenesis and host-directed therapy in HDV and HEV has not yet been published, but in the future, this novel technology could be used for modeling individual aspects of the hepatotropic D and E viruses.

#### 2.2.3. Cells Embedded in a Scaffold

Tran and collaborators introduced Huh-7 cell cultures in a calcium-alginate scaffold as a promising physiologically relevant model for the HCV infection. To achieve this, Huh-7 were cultured in calcium-alginate beads under dynamic conditions for 7 days. The results indicated a beneficial effect of the Huh-7-derived 3D culture in the calcium-alginate scaffold for producing a hepatic-like tissue expressing specific receptors to HCV and recapitulating the in vivo microarchitecture [90].

#### 2.2.4. A Microfluidic Chip System

Since a 2D static culture of PHH allows only short term studies, Temitope and collaborators established a microfluidic device using rat and human hepatocytes as a model for the HBV infection [91]. Besides, Kang and colleagues developed a microfluidic system of cocultured PHH and endothelial cells to recapitulate the sinusoidal microarchitecture. They successfully demonstrated that this microfluidic system could be used as a model for the HBV infection [92]. Ortega-Prieto and collaborators established a 3D microfluidic liver with PHH as a physiological platform for the HBV infection. This system extended the ex vivo maintenance of PHH for at least 40 days. To achieve this, they applied collagen-coated polystyrene scaffolds seeded with PHH, as well as microfluidic recirculation. The culture medium recirculation at a speed of 1μL/s provided the nutrients and oxygen to the cultures at stable levels, which is one of the main difficulties with the 2D PHH culture. This model could recapitulate the sinusoidal micropattern by functional canaliculi and cell polarization. They indicated that the 3D spheroid culture of PHH was a suitable model for viral infection studies. The MOI decreased by 10,000 times compared to 2D models. Moreover, cocultures of PHH and primary Kupffer cells provided clear evidence that Kupffer cells did not enable the HBV infection identification and did not cooperate in the early innate immune response. However, upon an exogenous stimulation, Kupffer cells rapidly secreted interleukin IL-6 and tumor necrosis factor-α, which could contribute to the observed suppression of HBV replication [13].

**Figure 1:**
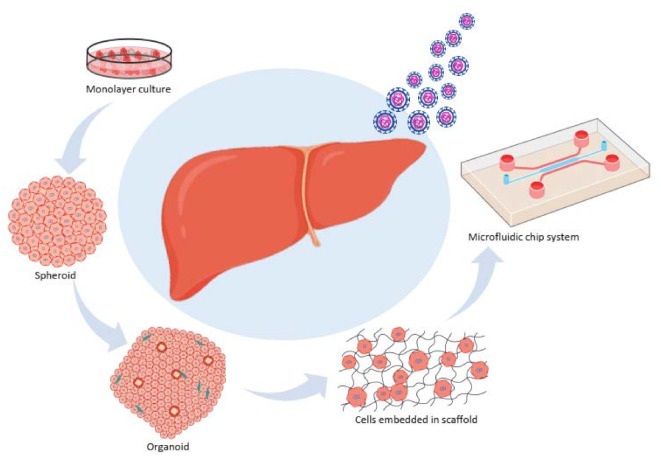
In vitro approaches in hepatotropic virus modeling.

Moreover, Ortega-Priet and colleagues developed a 3D liver-on-a-chip with PHH either in mono- or cocultivation with fibroblast cells. This microfluidic culture system created a great opportunity for long-term HBV infection with a physiological host cell response. In addition, the innate immune response and related cytokines were observed similar to those in HBV-infected patients [93]. However, not many research groups have directly applied these complicated system models in hepatotropic infections studies. These technologies propose potentially new insights into the host-pathogen interactions. In vitro approaches to study hepatotropic viral infection are shown in Figure 1 and Table 1.

## 3. Various In Vivo Approaches in Hepatotropic Virus Modeling

Studying hepatotropic viruses in vivo is limited because most of them are species-specific. For instance, HBV and HCV infect only humans, tree shrews, and some nonhuman primates [2,12].

The chimpanzee is a unique animal that is the closest one to humans and has remained a widely common model to study hepatotropic viruses for a long period of time. Despite the difficulties and costs of their handling, they have been successfully used to reveal the virus’s pathophysiology and enable the development of drugs and vaccines. For example, in experiments performed by Elmowalid et al., chimpanzees helped to reveal the specific cellular immune response that occurred after their immunization with recombinant HCV-like particles [94]. Using chimpanzees, Forns et al. showed that the vaccine based on DNA encoding cell-surface expressed E2 glycoprotein could prevent the infection progression [95]. Lanford et al. revealed that an agonist of toll-like receptor 7 can decrease viremia in HBV-infected animals [96]. Nevertheless, due to ethical issues, nowadays, most countries have already banned using nonhuman apes for experiments.

Tree shrews, also known as Tupaia belangeri, were proven to be susceptible to both HBV [97] and HCV [98]. Despite their low rates of captive breeding and the lack of specific reagents [12], tree shrews remain of interest in the studies on hepatotropic viruses. For instance, this model allowed scientists to reveal a functional HBV and hepatitis D virus (HDV) receptor NTCP [50]. Moreover, compared to chimpanzees, tree shrews can also suffer from HBV-associated hepatocellular carcinoma that makes them promising for the studies on this condition [99].

Due to the lack of animals receptive to human hepatotropic viruses, another approach is applied to study homological viruses: Woodchuck hepatitis B virus (WHBV) [100], duck hepatitis B virus (DHBV) [101], deer mouse hepatitis C virus [102], etc. Particularly, Menne et al. showed that the combination of RG7834 (inhibitor of HBV expression), entecavir, and interferon-α significantly decreased viremia in WHBV chronic infection, but the effect was not sustained [100]. Nevertheless, despite the possible benefits, the species-determined differences may cause significant issues.

Thus, the most promising approach to study human hepatotropic viruses is using genetically modified animals (particularly mice), which will be discussed further in more detail.

### 3.1. Transgenic Animals

**Figure 2:**
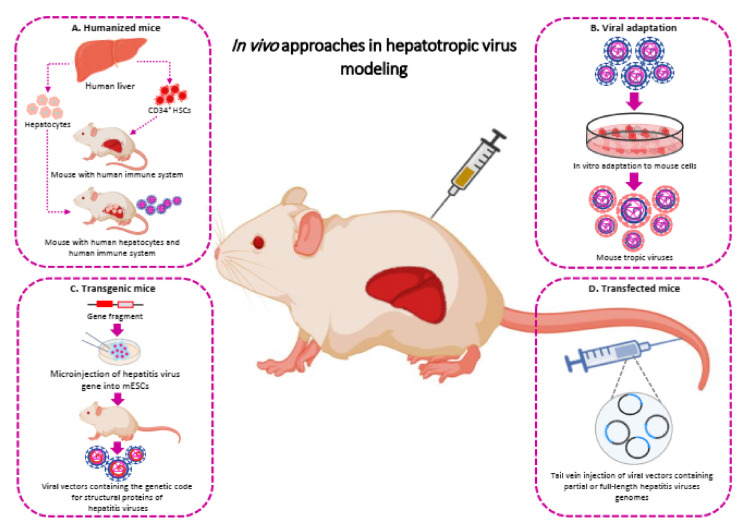
Overview of the main current in vivo approaches used to study hepatotropic virus infections. (**A**) Humanized mice. Human cells (HSCs and hepatocytes) are injected into the spleen and portal veins of uPA/RAG-2, uPA/SCID, TK-NOG, and FRG mice. These cells migrate to the liver via the splenic and portal veins, engraft into the liver structure, and proliferate. (**B**) Viral adaptation. In vitro adaptation of hepatotropic viruses to mouse cells, may adapt these viruses to the murine environment. (**C**) Transgenic mice. Microinjection of structural viral genes into mESCs used to develop HBV/HCV transgenic mice. (**D**) Transfected mice. This model can be constructed by hydrodynamic injection and adeno-associated viral transfection of viral vectors containing partial or full-length hepatitis virus genomes. Abbreviations. FRG, Fumaryl acetoacetate hydrolase (Fah)/RAG2/interleukin (IL) 2-gammaC (FRG) triple mutant mice; HSCs, Hematopoietic stem cells; mESCs, Mouse embryonic stem cells; TK-NOG, a NOG mouse expressing a thymidine kinase transgene (NOG-Tg(Alb-UL23)7-2/ShiJic); uPA/RAG-2, urokinase-type plasminogen activator (uPA)/recombinant activation gene-2 (RAG-2).

To date, various transgenic animal models have been offered to study hepatotropic viruses. They can be divided into two main groups: Animals expressing single antigens or the full viral genome and humanized animals (Section “Humanized animals”) (Figure 2C). Animals from the first group usually include those expressing HBsAg [103], HbcAg [104], HbeAg [105], HBx [106], HCV envelope genes [107], HCV core [108] or the full HBV or HCV viral genome [109,110,111,112].

Although transgenic models (usually murine ones) carrying viral genes do not recapitulate several crucial stages of the virus pathogenesis (viral entry, nuclear import, and cccDNA formation), they ensure the virus replication and secretion of viral particles [2], and therefore, can contribute most to the studies on drugs influencing these particular stages. For instance, such models were used to reveal the antiviral activity of lamivudine and adefovir dipivoxil [113], entecavir [114], and polyoxometalate [115] against HBV. Likewise, Tokunaga et al. showed the significant antifibrotic effect of PRI-724, a selective inhibitor of β-catenin/CBP, using HCV-transgenic mice [116]. However, such models can also be applied to reveal the features of the virus pathogenesis. For example, Satoh et al. showed that natural killer (NK) cells participated in eliminating core-expressing hepatocytes during the acute phase of HCV infection [117]. Using a transgenic mouse, Chouteau et al. revealed that the HCV protein expression in hepatocytes contributed to the development of hepatic fibrosis in the presence of other inducing agents [118].

Nevertheless, mice are not a single species used to establish transgenic models suitable to study hepatotropic viruses. Particularly, using a transgenic zebrafish, Lu et al. demonstrated that aflatoxin B1 and HBx had a synergistic effect on both the lipid metabolism regulation and the cell cycle division that promoted steatosis and hyperplasia, respectively [119].

### 3.2. Humanized Animals

As mentioned above, most hepatotropic viruses are species-specific; so, the development of humanized animal models is in high demand. Such animals carry a human genetic material that makes them artificially susceptible to human hepatotropic viral infection (Figure 2A).

One of the first humanized models used in HBV/HCV studies was the ectopic transplantation of human liver tissue under the kidney capsule. This method ensures susceptibility to HCV and HBV, and HDV superinfectivity [120,121]. However, it has low productivity (low virus titer in the blood) and cannot be used for long-term experiments because of the short-lasting viability of the transplanted liver tissues. One of the approaches to overcome these obstacles is to create a native-like environment for human hepatocytes inside an animal body that can be achieved by replacing mouse hepatocytes with human ones. Thus, the induced death of the mouse liver is followed by the transplantation of human cells, which migrate to the liver via the splenic and portal veins, engraft, start to proliferate, and integrate into its structure.

However, humanized animal models can also be created using genetic tools. One of the first such models was an immunodeficient urokinase-type plasminogen activator (uPA)/recombinant activation gene-2 (RAG-2) (uPA/RAG-2) mouse repopulated with human hepatocytes [122]. In that study, the liver of homozygous uPA/RAG-2 mice was damaged, and the animals were intrasplenically injected with human hepatocytes to restore their functioning. However, this procedure caused a relatively high death rate after the manipulations and showed low human hepatocytes’ engraftment. Moreover, the liver of all the treated mice did not functionally recover [122]. Some scientists claim that such failures are mostly caused by unoptimized protocols to prepare liver tissues. For instance, properly isolated tupaia hepatocytes were successfully engrafted and repopulated the liver of uPA/RAG-2 mice [123]. Although the uPA mutation facilitates the engraftment of human hepatocytes, the transplanted cells are often damaged by native liver parenchymal cells. Moreover, the breeding of such mice does not have high efficacy, they usually have renal diseases, and the time for transplantation of hepatocytes to prevent animal death is significantly limited [124]. Despite the mentioned drawbacks, this approach is used to model hepatotropic viral infections, particularly, HBV, and test antivirals as a part of preclinical trials [125] because it enables the recapitulation of subviral particles and virions in sera similar to those in humans and HBV-related structures within the infected cells [122,126]. The modification of this model—uPA/ severe combined immunodeficiency (SCID) mutated mice—is an established platform to study the HEV infection and HBV/HDV coinfection and superinfection [127,128]. A number of antivirals, such as griffithsin, ribavirin, and pegylated interferon alpha, have been proven to be effective against hepatotropic viruses with using this model [129,130]. uPA/SCID murine model has been shown to be helpful in the study of the HEV pathogenesis, particularly viral shedding that was similar to that in humans [129,131]. It was revealed that stool-derived HEV had higher infectivity than plasma-derived virions because of the absence of the envelope, higher RNA, and lower ORF2 Ag content [132].

Another line widely used for modeling is fumaryl acetoacetate hydrolase (Fah)/RAG2/interleukin (IL) 2-gammaC (FRG) triple mutant mice. In contrast to uPa-mutated mice, the liver injury of such animals can be controlled with 2-[2-nitro4-(trifluoromethyl)benzoyl] cyclohexane-1,3-dione (NTBC) that prevents the accumulation of toxic tyrosine catabolites, due to the lack of Fah [133]. Moreover, modeling that uses such animals can be performed with populations of any age (not only newborn mice) and includes serial cell transplantation; they do not suffer from renal diseases and demonstrate relatively high levels of human hepatocyte chimerism [134]. Such animal models are susceptible to the hepatotropic viruses and maintain the HBV, HCV, and HEV infections [135]. Thus, they are feasible for drug screening that has been proven in several studies. Particularly, such HCV-infected mice positively responded to the treatment with PEGylated interferon α 2a, a nucleozide analog, ribavirin, a cyclophilin inhibitor, Debio 025, and a nucleotide analog, adefovir dipivoxil [136]. FRG mice were used to assess the remodeling of T-cells as a novel anti-HBV therapy [137]. Additionally, when infected with the HEV-containing human plasma, these chimeric mice may be applicable to study HEV blood transmission [135]. FRGS mice—a modification of FRG mice—were also shown to be feasible, and particularly, were used to study human-induced pluripotent stem cells as a novel hepatocyte source for engraftment [138]. Nevertheless, FRG mice can suffer from liver carcinomas, due to the Fah-deficiency, and require drug treatment during long-term experiments that can significantly affect the study results [139].

The establishment of TK-NOG severely immunodeficient mice expressing herpes simplex virus type 1 thymidine kinase has enabled the repopulation of human hepatocytes without the need for administration of exogenous drugs. A single ganciclovir injection can induce a liver injury [124], and this animal model is more susceptible to the HBV and HCV infection than those mentioned above [140]. It was used to reveal the fundamentals of the virus interactions with host cells [141,142], and to test antivirals, such as entecavir and interferon, and drug candidates, such as modified lamivudine [143,144].

Since the models described above are immunodeficient, neither pathogen- or drug-induced immune responses can be investigated. Thus, such models cannot be applied to study the immunopathology of hepatotropic viral infections.

To study these aspects, several approaches have been offered. One of them uses AFC8 chimeric immune mice. Compared to those mentioned above, AFC8 mice undergo the transplantation of both human hepatocytes and CD34^+^ hematopoietic stem cells [145,146]. As a result, the immune response to the HCV infection (immune cells infiltration, T-cell expansion, and synthesis of inflammation factors) was similar to that in humans. However, this model, like the previous ones, fails to recapitulate the adequate B-cell response and secretion of specific antibodies. Unfortunately, to date, no additional studies using this model have been published, probably, because of the relatively low repopulation efficacy (only 15%). Another approach is based on the breeding of immunodeficient mice with normal ones. Thus, animals, such as TK-NOG mice, can be successfully used to achieve a competent immune generation [143]. Moreover, FRGS mice have also been reported to be used as a platform to create immune dual-humanized mice to study hepatotropic viral infections [147]. Yuan et al. showed that this model was susceptible to the HBV infection leading to liver cirrhosis and possessed a specific immune response: The increased production of human NK cells, macrophages, T-cells, and human cytokines, such as IL-6, IL-17, IL-4, and IL-10. Probably, the most adequate murine model to study hepatotropic viral infections in vivo was proposed by Dusséaux et al. [148]. They created BALB/c *Rag2^–/–^Il2rg^–/–^Sirpa*^NOD^Alb-uPA^tg/tg^ mice with engrafted human hepatocytes and immune cells. Such a model can reproduce the full HBV cycle and produce HBV-specific antibodies, similar to humans. Thus, solving the immune system-related issues of humanized animal models would provide new insight into hepatotropic viral infections.

### 3.3. Viral Adaptation

Although the studies on the viral adaptation are limited and the efficacy of this approach is still unclear, it can be of interest to reveal the fundamentals of pathogen-host interactions and host-related features (Figure 2B).

While infecting a nonspecific host, viruses usually stop their life cycle at certain stages. For instance, mouse cells can maintain HCV replication, but new virions are not released from them [149,150]. One of the factors blocking the HCV life cycle in nonhuman cells is the absence of particular proteins responsible for its entry: Tetraspanin CD81, scavenger receptor class B type 1, claudin-1, occludin, etc. [58,151,152]. The increased efficacy of the HCV entry into murine cells was shown to be achieved in vitro using viral adaptation to mouse CD81 by mutations in HCV glycoproteins [153]. The infectivity of such CD81-adapted murine tropic HCV was also revealed in in vivo experiments using the uPA/SCID murine model [154]. However, in both cases, the infectivity was low, and the persistent infection still was not reached. Better results were achieved using HCV adapted to nonhuman primates: Serum viremia in humanized mice was observed for up to 10 weeks [155]. Further research on viral adaptation is warranted, to establish new animal models required for drug and vaccine development.

### 3.4. Transfected Models

### Hydrodynamic Injection and Adenovirus (AdV)

Adeno-associated viral systems have also been used for the delivery of hepatotropic viruses into murine hepatocytes. Hydrodynamic injection of viral vectors containing partial or full-length hepatitis viruses’ genomes is an efficient technique to deliver the genetic material in the liver of mice [151]. This approach as a nonviral method involves a rapid injection of a large amount of liquid containing naked DNA into the tail vein of animals, which results in the uptake of the viruses’ genomes in the liver and blood cells and an adaptive immune response that clears the hepatitis infection (Figure 2D) [152]. In comparison with transgenic models, hydrodynamic injection is more adaptable and has been used to study replication of distinct HBV genotypes and to evaluate drug resistance using reverse genetics. Moreover, this approach has been used to establish HBV, HCV, and HDV models in mice [156]. Besides, it is appropriate for the study of immune responses during acute or chronic hepatitis and prospective immunotherapeutic interventions in treating liver disease. It can also be used in the identification of different hepatitis viruses’ genotypes and subtypes in vivo, and in the screening of antiviral compounds [157]. HBV replicative DNA, genomic RNA, and proteins were detected for up to three months after an intravenous injection of AdV-HBV. This method can lead to efficient hepatitis B virus’s replication in in vitro and in vivo models [158].

Some studies have indicated that transfected models can induce innate [159] and adaptive immune responses in mice [160], and diverse effector cells, including natural killer cells, CD4+ T-cells, and CTLs (CD8+ T-cells), are essential for the clearance of the viral transcriptional template from the liver in these models [161,162,163].

Gao and colleagues investigated the effects of interleukin-33 in an HBV mouse model. Interleukin-33 inhibited HBV via the ST2 receptor in the HBV mouse model. The results have shown that NK cell-derived antiviral cytokines may have a critical role in the early viral clearance during the HBV infection [164]. One study investigated the effects of monocyte chemotactic protein-induced protein 1 (MCPIP1) as a potential therapeutic target for the HBV infection. Hydrodynamic tail vein injection was used to transfect pHBV1.3. In some experiments, pHBV1.3, together with human MCPIP1-pcmv/tag2a were injected. The obtained data showed that MCPIP1 significantly suppressed HBV replication by degrading viral RNA, and inhibited HBV-induced proinflammatory cytokines [165].

Several factors, including a plasmid backbone, mouse strain, age, and sex, can affect the level and duration of replication of the hepatitis viruses. The use of a lentiviral backbone instead of an AAV vector led to increased and prolonged HBV replication [161,166]. HBV replication continuously lasted for up to six months after a hydrodynamic injection of the pAAV/HBV1.2 plasmid into C57BL/6 mice [167]. In another study, persistent viral replication (up to 46 weeks) was reported with using pAAV/ HBV1.2 plasmids injected into C3H/HeN mice [162]. HBV is cleared rapidly from BALB/cJ and NOD/ShiLtJ mice [163]. Moreover, Dion and colleagues developed a chronic HBV infection model in which the livers of mice were transduced with adeno-associated virus serotype 2/8. These mice had virological and immunological features of chronic HBV infection, including hepatitis B virus e antigen, hepatitis B virus surface antigen, and the presence of HBV DNA in the serum for at least one year. Thus, this murine model could be useful for the development of new therapeutic strategies for chronic HBV infections [168]. A study used a transfected mouse model for evaluation of the immune response to the HBV infection. The obtained results indicated that a follicular helper T-cell-response to HBsAg was necessary for the HBV clearance, and deterioration of these cells promotes chronic HBV in mice and humans [169].

Transfected mouse models can be used for the evaluation of broad-spectrum antiviral compounds for liver disease. Several studies used small interfering RNA (*siRNA*) and short hairpin RNA (*shRNA)* to minimize the HBV and HCV expression and liver failure. For instance, McCaffrey and colleagues cotransferred plasmids expressing shRNAs homologous to HBV mRNAs into mice with HBV to reduce the replicated viral genomes, viral RNAs, and secreted HBV surface antigen (HBsAg) [170]. In another study, Wu and colleagues transferred a shRNA-expressing plasmid, pSuper/HBVS1, to HuH-7 cells and mice. This shRNA knocked down the expression of a conserved region of HBV DNA and remarkably decreased levels of viral RNA and proteins [171]. Thus, such an approach could be useful in treating HBV. Furthermore, a model of hepatitis C was developed by Kim and colleagues. They transferred HCV-core-specific siRNA to mice with HCV using a delivery system based on apolipoprotein A-I-bound cationic liposomes. The results indicated a decrease in the viral gene expression in the liver, two days after the injection [172].

A promising novel approach is the use of the HBV-specific clustered regularly interspaced short palindromic repeats (CRISPR)/Cas9 system for selective elimination of cccDNA and the viral protein. In hydrodynamic-based mouse models, using HBV-specific guide RNA (gRNAs) efficiently inhibits levels of HBV-expressing vectors and facilitates the clearance of the intrahepatic HBV without any toxicity [173,174].

Huang and colleagues used a transfected mouse model to evaluate the therapeutic effect of hepatitis delta antigens (HDAg) in the HDV infection. The results showed that the Tat-enhanced delivery of the C terminus of the HDAg-L (TAT-HA-HDAg L (198–210)) fusion protein suppressed the viral particles’ assembly and secretion of HDV and could be used as a new therapeutic compound against the HDV infection [175].

The most significant benefit of hydrodynamic injection is the possibility to investigate the immune responses during the acute phases of infections which appear when viral sequences are not integrated into the host genome. Another privilege of this system is that the replication of HBeAg or HBsAg seroconversion proceeds without the manifestation of obvious signs and symptoms of liver diseases [168]. Most transfected mouse models that study the hepatotropic viruses are based on the HBV, HCV, and HDV infection, and transfected mice for the hepatotropic viruses A and E are still under development. Hereafter, this animal model could be used for modeling individual aspects of these hepatotropic viruses. In vivo approaches to study hepatotropic viral infections are shown in Figure 2 and Table 2.

## 4. Conclusions

Various model systems have been established for modeling individual aspects of the hepatotropic viruses’ life cycle or the complete infection cycle in vivo and in vitro. Since each system has its benefits and caveats, selecting the best model is a challenging task. Today, many biomedical studies depend on one of the two approaches, either 2D cell culture experiments or animal models. However, these systems have some drawbacks. Cells cultivated in a 2D culture have been shown to follow different adaptations in terms of gene expression patterns and cell physiology, as compared to those grown under 3D conditions. Studying the hepatotropic viruses in animal models is limited because most of them are species-specific. Moreover, conducting research with animal models in this field raises ethical concern. There are several limitations to the use of humanized liver in chimeric mice, including their immunodeficient background, pathogenesis, and immune response, which hamper their broad application [176]. Thus, further development of more sophisticated infection models is inevitable. The most promising approach to the studies of human hepatotropic viruses could be using genetically modified animals. In parallel, 3D culture systems, including spheroids and organoids, have received remarkable attention in modeling hepatotropic viral infection. However, each model system is expected to address a specific question. Thus, the confirmation of the results with different systems should be encouraged.

**Table 1:** Summary of various tissue culture models for hepatotropic viruses.

Culture Model	Cell Type	HAV	HBV	HCV	HDV	HEV
2D	Primary cells		Adult PHH [15,21]	Adult PHH [17]	Adult PHH [18,19,20,21]	Adult PHH [18] primary human intestinal cells [23] primary human endometrial cells [24]
	Cancer cell lines		HepAD38 [34] HepDE19 [35] Hepa RG [44,46] HLCZ01 [36] (HepNB2.7) [38] HrpaRG [39,40] HepG2 +Huh-7 [49,55] HepG2 [51] HepG2-NTCP [52] HLC [24,26,64]	HLCZ01 [36] HepG2 [51] Huh-7 [55]	RG [45,46] HepG2, Huh-7 [50]	HepaRG cell line, PICM19 [43] Hepa RG [44] HepG2 and Huh-7 [53] HepG2 and HepG2/C3A [54]
	PSCs			HLC [61,62,63,66,67,68,69]		HLC [70,71,72]
	Co-culture		Adult PHH + Murine fibroblast cells [28], Fetal PHH + nonparenchymal cells [29], Adult PHH + Murine stromal cells [30]	Adult PHH + Murine fibroblast cells [26], Adult PHH + Murine fibroblast cells [28]	PHH + 3T3-J2 [31]	peripheral blood mononuclear cells + bone marrow-derived macrophages [32] primary proximal tubular epithelial cells + immune cells [33]
3D	Spheroid	HLCs [91]	HCC cell lines + fibroblast cells + HUVEC + stellate cells [83]	Adult PHH [75] Immortalized PHH [76] Huh-7 [77,78,79] Huh-7.5 + PHH [81]		
	Organoid					
	Scafffold based		PSC-endoderm+ mesenchymal stem cells +HUVEC [87] HCC-derived PHH/Healthy PHH [176] HepLPCs [82]	Huh-7.5 [89,90]		
	Microfluid chip		Rat hepatocyte, PHH [93] PHH+endothelila cells [94] PHH with/without murin fibroblast cells [95]	Huh-7 + calcium alginate scaffold [92]		

**Table 2:** Animal models are used to study hepatotropic viruses.

Animal	*N*	Infecting Agent	Therapeutic Agent	Outcomes	Ref.
Chimpanzee	4	HCV	Recombinant HCV-like particles and AS01B adjuvant	HCV-specific cellular immune response induced by immunization	[96]
Woodchuck	27	WHBV	RG7834 in combination with ETV and IFN-α	Efficacy of the treatment No sustained antiviral response	[102]
Transgenic HBV mice	113	HBV	ETV	Minimal effective daily doses for male and female mice revealed	[116]
Transgenic HCV mice	21	HCV	PRI-724	Significant antifibrotic effect	[118]
uPA/RAG-2 mice	25	WMHBV	HBVpreS/2-48^stearoyl^ HBVpreS/2-39^myr^	Inhibition of virus entry by peptides	[127]
uPA/SCID mice	6	HBV and HDV	Myrcludex-B	Inhibition of HDV infection In vivo kinetics of HDV spreading revealed No detectable HDV and HBV serological markers in treated mice	[129]
uPA/SCID mice	24	HEV	PEGylated IFN-α	Rapid viral clearance Confirmation of model applicability [132]	
FRG mice	12	HCV	PEGylated IFN-α 2a, ribavirin, Debio 025, adefovir dipivoxil	Same efficacy of the treatment with different agents	[137]
FRG mice	9	HEV	-	Model susceptibility [138]	
FRGS mice	6	HBV	Myrcludex B and ETV	Drug synergism Reduced HBV RNA and DNA production	[141]
TK-NOG mice	8	HBV	NM23TC	Sustained anti-HBV response	[146]
AFC8 mice	6	HCV	-	Infiltration with human T-cells, macrophages, and dendritic and NK cells observed Human HCV-specific T-cell response Poor B-cell response	[147]
BALB/c Rag2^−/−^ IL-2Rγc^−/−^ NOD.sirpa uPA^tg/tg^ mice	4	HBV	ETV	Immune cells infiltration observed Synthesis of HBV-specific IGs Virus progression affected by immune system	[150]

Abbreviations: ETV, entecavir; FRG, fumaryl acetoacetate hydrolase (Fah)/RAG2/interleukin (IL) 2-gammaC; FRGS, Fah^−/−^Rag2^−/−^IL-2Rγc^−/−^SCID; HEV, hepatitis E virus; HBV, hepatitis B virus; HCV, hepatitis C virus; IFN, interferon; IG, immunoglobulin; NK, natural killer; NM23TC, nanoformulation of the modified lamivudine 3TC; PRI-724, selective inhibitor of β-catenin/CBP; RG7834, inhibitor of HBV expression; uPA/RAG-2, urokinase-type plasminogen activator/recombinant activation gene-2; uPA/SCID, urokinase-type plasminogen activator/severe combined immunodeficiency; WHBV, woodchuck hepatitis B virus; WMHBV, woolly monkey hepatitis B virus.

## Data Availability

Not applicable.

## Abbreviations

2D	Two dimensional
3D	Three dimensional
AdV	Adenovirus
** c ** ccDNA	Covalently closed circular DNA
FRG	Fumaryl acetoacetate hydrolase (Fah)/RAG2/interleukin (IL) 2-gammaC (FRG) triple mutant mice
HAV	Hepatitis A virus
HBV	Hepatitis B virus
HCC	hepatocellular carcinoma
HCV	Hepatitis C virus
HDV	Hepatitis D virus
HepG2	Hepatoma G2
HLCs	Hepatic like cells
HUH7	human hepatocellular carcinoma cell line 7
HUVECs	Human umbilical vein endothelial cells
IL	Interleukin
NK	Natural killer
NTCP	Sodium taurocholate cotransporting polypeptide
PHH	Primary human hepatocyte
pgRNA	Pregenomic RNA
PSCs	Pluripotent stem cells
SCID	Severe combined immunodeficiency
* shRNA *	Short hairpin RNA
* siRNA *	Small interfering RNA
TK-NOG	NOG mouse expressing a thymidine kinase transgene (NOG-Tg(Alb-UL23)7-2/ShiJic)
uPA/RAG-2	urokinase-type plasminogen activator (uPA)/recombinant activation gene-2 (RAG-2)
WHBV	Woodchuck hepatitis B virus
